# Editorial: Oro-facial pain: pathophysiology, molecular mechanisms, diagnostic innovations and multidisciplinary management

**DOI:** 10.3389/froh.2026.1855968

**Published:** 2026-05-19

**Authors:** Ruwan Duminda Jayasinghe, Rasika Manori Jayasinghe, Dhanushka Leuke Bandra, Kehinde Kazeem Kanmodi

**Affiliations:** 1 Department of Oral Medicine and Periodontology, University of Peradeniya, Peradeniya, Sri Lanka; 2 School of Health and Life Sciences, Teesside University, Middlesbrough, United Kingdom; 3 Department of Prosthetic Dentistry, University of Peradeniya, Peradeniya, Sri Lanka; 4 Department of Prosthetic Dentistry, Mahsa International College (Penang), Butterworth, Malaysia; 5 Centre for Dental and Craniofacial Research, Cephas Health Research Initiative Inc., Ibadan, Nigeria; 6 Department of Public Health, Thomas Adewumi University, Oko, Nigeria; 7 Department of Research, University of Technology and Entrepreneurship, Phnom Penh, Cambodia

**Keywords:** diagnostic innovations, multidisciplinary management, oral health-related quality of life, oro-facial pain, precision medicine

Orofacial pain (OFP) encompasses a diverse group of disorders which poses a significant clinical challenge, often progressing from acute to chronic conditions due to a disconnect between clinical diagnosis and underlying pathophysiology (1). OFP is clinically complex in both diagnosis and management and is due to multifactorial etiologies, including musculoskeletal, neurological, behavioral and psychosocial factors resulting in unclear etiopathogenesis at times. In addition, complex anatomical innervation by the trigeminal nerve, overlapping pain referral patterns, multiple tissue origins and chronic nature complicate it further, resulting, oftentimes, for the need of multidisciplinary care due to common involvement of other comorbidities like anxiety, depression, and poor sleep. OFP cannot be completely differentiated from pain arising in other areas of body and at times it is strongly connected with other pathologies. A strong association between OFP and oral health-related quality of life (OHRQoL) has been well documented, and its effect is more related to sleep, emotional stability, and performing daily tasks (2). As OFP is not a single disease but a disease of multiple types mainly characterized into nociceptive pain, neuropathic pain, and nociplastic pain; hence, its management requires combined treatment modalities to address the diverse underlying causes and manifestations (3, 4).

Even though a significant number of research papers have been published on OFP, still a large knowledge gap exists. Therefore, this special issue titled “Oro-facial Pain: Pathophysiology, Molecular Mechanisms, Diagnostic Innovations and Multidisciplinary Management” was initiated with the aims to bridge the existing gaps in knowledge by highlighting cutting-edge research on the pathophysiology and molecular mechanisms underlying OFP, while further exploring innovative diagnostic tools and interdisciplinary management strategies. This was to act as a platform to highlight the integration of research in basic science and clinical practice for translational research.

OFP significantly impacts the quality of life of the affected individuals and therefore treating patients with OFP successfully is of utmost significance. Multi-disciplinary research is essential in filling up the existing gaps. Despite the management options, both pharmacological and non-pharmacological available, treating OFP is a challenge due to heterogeneity of different pain conditions and inter-patient variations observed in patients with OFP. Accurate diagnosis of the disease is crucial as incorrect diagnosis can lead to under or over treatment. Overlapping symptoms between different anatomical components is not uncommon leading to difficulties in diagnosis. Accurate assessment is essential as OFP can be involved with conditions with serious consequences like temporal arteritis and fibromyalgia or can mimic serious conditions like cancer pain. Also, its diagnosis is always a challenge due to subjectivity, lack of biomarkers and due to delayed presentation. Further studies are required to develop an evidence based diagnostic workflow.

Multiple new areas are under investigation to identify the exact etiopathogenesis of the OFP, which plays a key role in proper management of the condition. Genetics are one of the areas that has taken a center stage with key interest among researchers. The genetics of OFP can influence each and every step of OFP including pain initiation, pain processing, pain perception, inflammatory responses, connective tissue integrity, and neural repair mechanisms. Based on the individual patient's genetic profile, precision medicine, a new concept which is also referred to as personalized medicine or personalized care has emerged. This may result in paradigm shift in the management of untreatable chronic OFP conditions (3).

New cells have also been identified to play a role in the pain process in addition to the well-known anatomical structures. Overall, the addition of this new knowledge, which is reported in this special issue, will shed light to the understanding of OFP mechanisms allowing the patients to have a good quality of life. Clinicians need to adopt these newer findings to their management strategies expanding the evidence-based management of patients.
